# Supplementary figures and images for: Distribution Map of Peristaltic Waves in the Chicken Embryonic Gut Reveals Importance of Enteric Nervous System and Inter-Region Cross Talks Along the Gut Axis
Source: Front Cell Dev Biol. 2022 Feb 4;10:827079. doi: 10.3389/fcell.2022.827079 (PMC8874353; doi:10.3389/fcell.2022.827079)

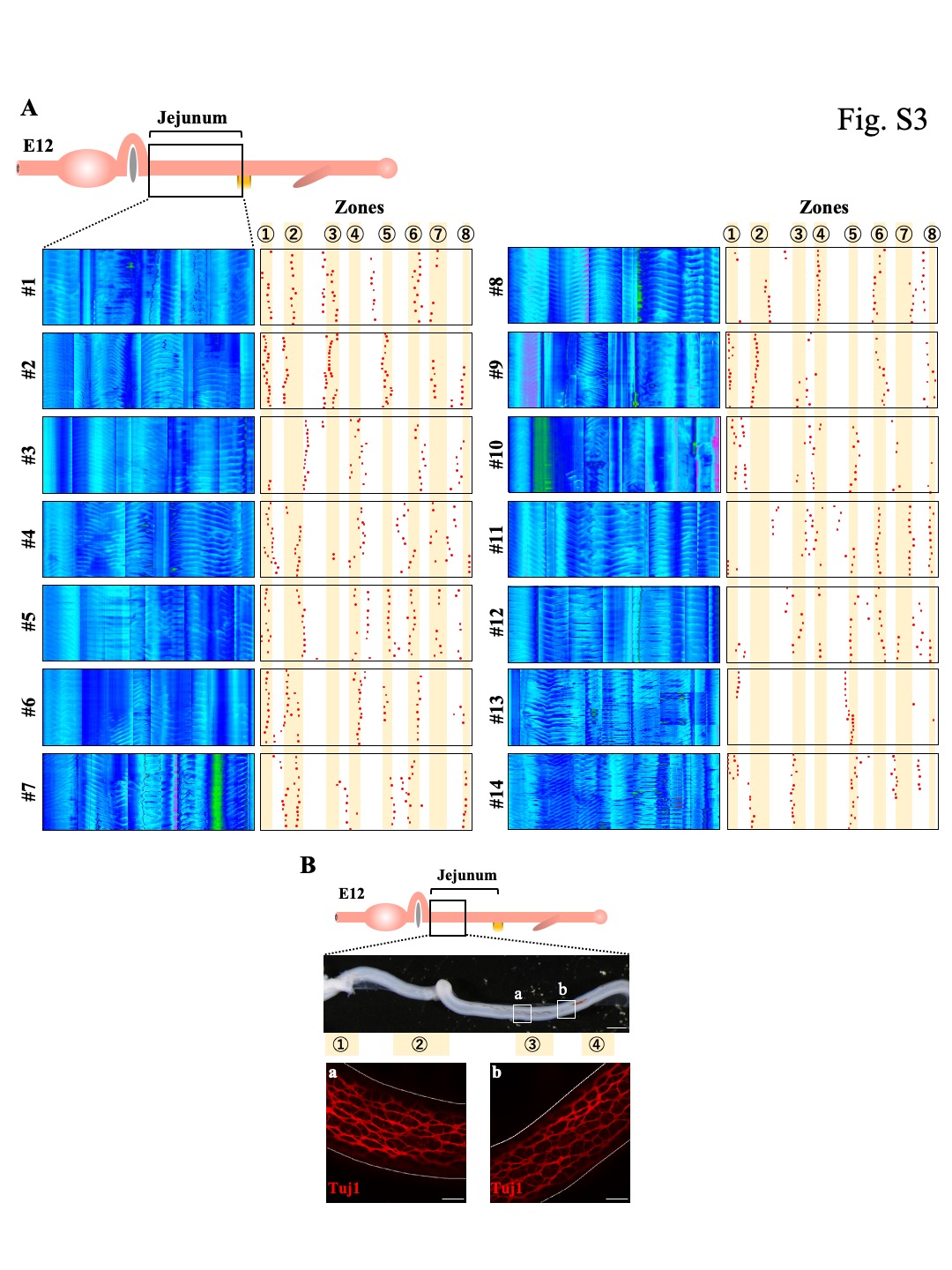

Supplement: Supplementary file 1 [file Image3.JPEG]

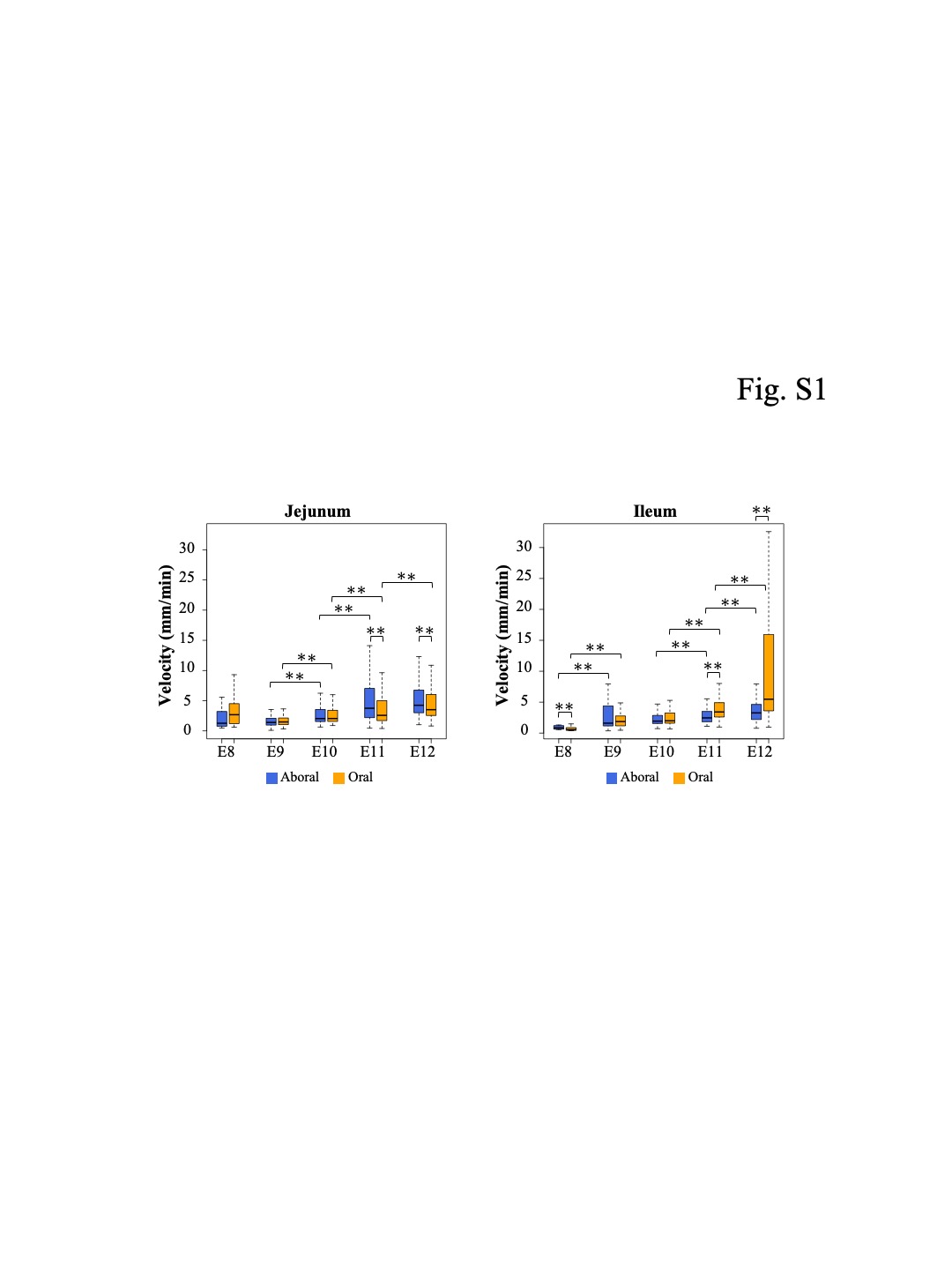

Supplement: Supplementary file 3 [file Image1.JPEG]

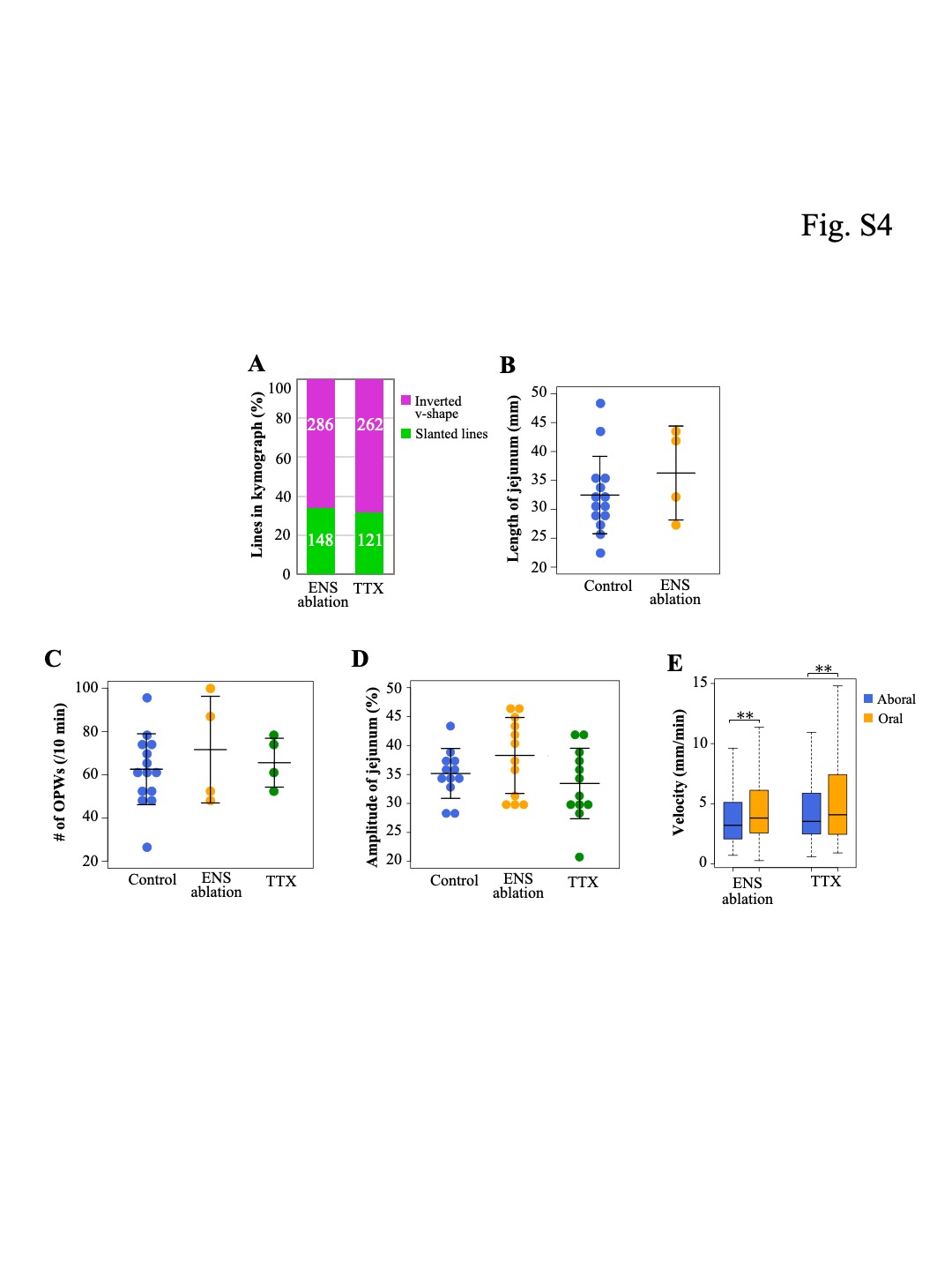

Supplement: Supplementary file 4 [file Image4.JPEG]

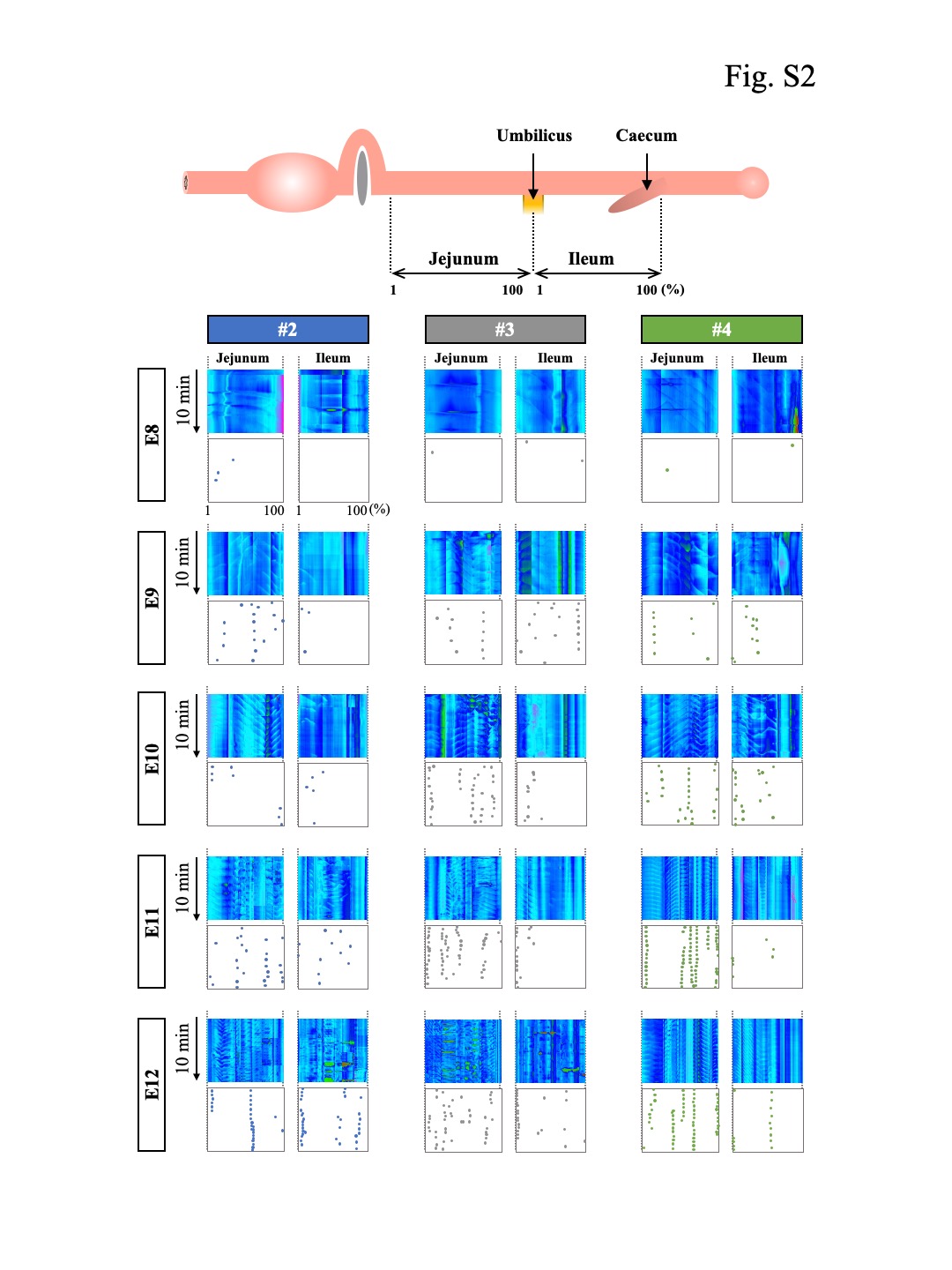

Supplement: Supplementary file 5 [file Image2.JPEG]

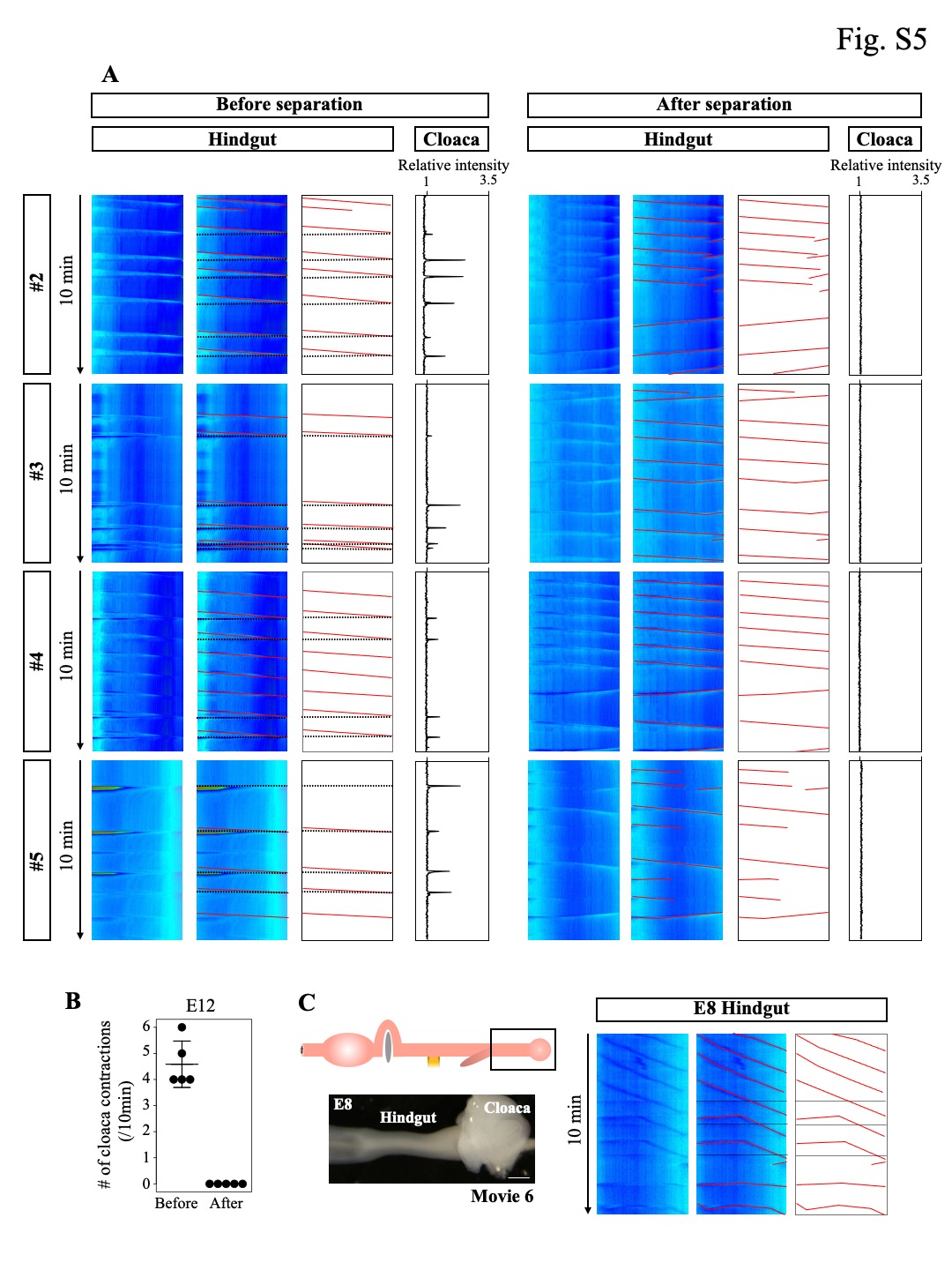

Supplement: Supplementary file 6 [file Image5.JPEG]

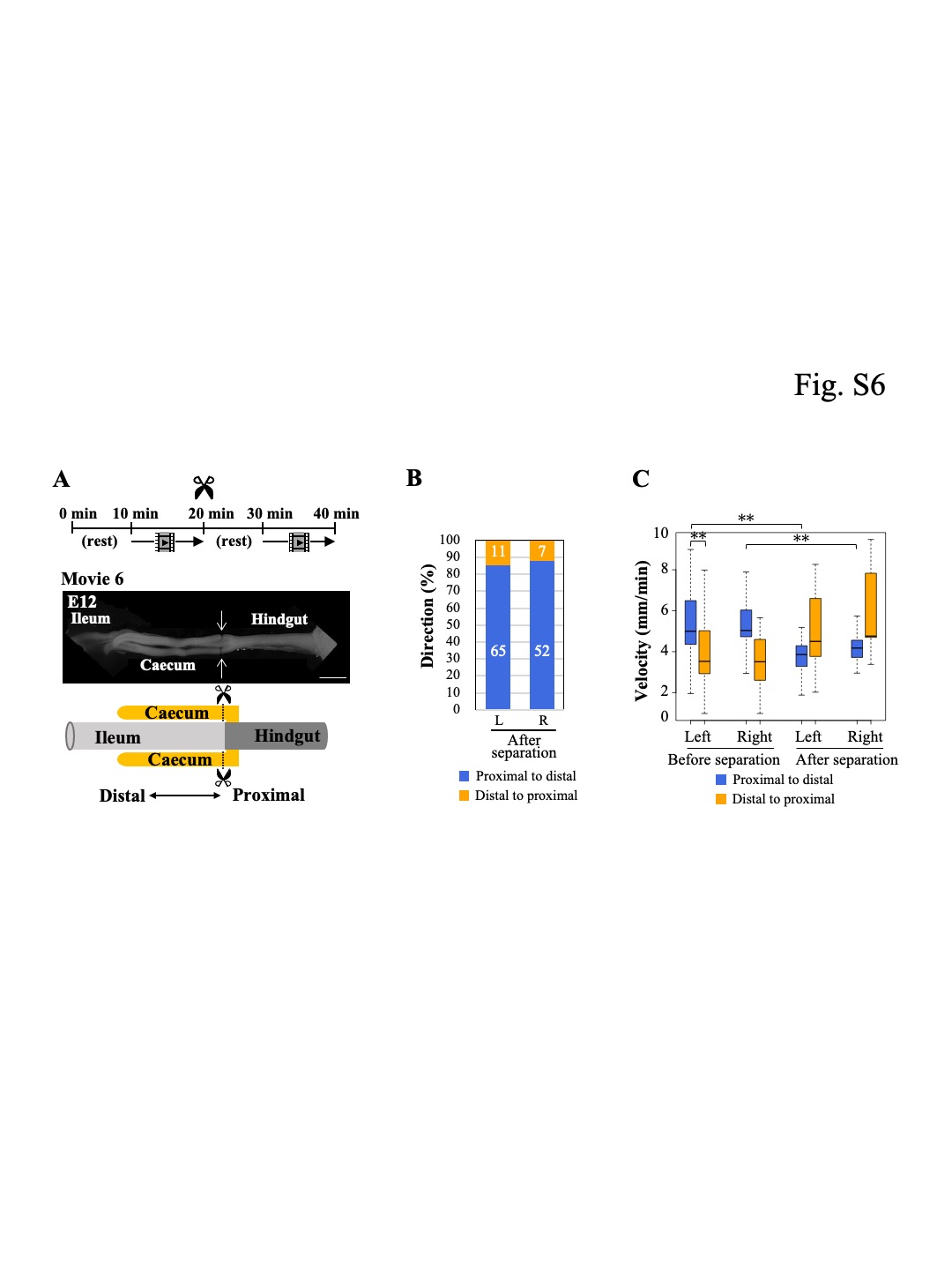

Supplement: Supplementary file 13 [file Image6.JPEG]
